# Temporo-Mandibular Joint Functional Arthroplasty: Does It Improve the Short-Term Quality of Life in Patients with Painful Anterior Disc Displacement Without Reduction? A Retrospective Cohort Study

**DOI:** 10.3390/jcm14082560

**Published:** 2025-04-08

**Authors:** Fabrizio Spallaccia, Silvia De Tomaso, Giulio Cirignaco, David Faustino Angelo, Luigi Angelo Vaira, Valentino Vellone

**Affiliations:** 1 Department of Maxillofacial Surgery, “S. Maria” Hospital, 05100 Terni, Italy; 2 Department of Maxillofacial Surgery, University of Siena, Policlinico Santa Maria alle Scotte, Viale Mario Bracci, 53100 Siena, Italy; 3 Faculdade de Medicina da Universidade de Lisboa, Avenida Prof. Egas Moniz, 1649-028 Lisboa, Portugal; 4 Maxillofacial Surgery Operative Unit, Department of Medicine, Surgery and Pharmacy, University of Sassari, 07100 Sassari, Italy; 5 Department of Life Science, Health and Health Professions—Università degli Studi “Link”, 00165 Rome, Italy

**Keywords:** temporomandibular joint, functional arthroplasty, quality of life

## Abstract

**Background**: Anterior disc displacement without reduction (ADDwoR) of the temporomandibular joint (TMJ) often leads to persistent pain and reduced quality of life (QoL). Conservative treatments frequently fail to provide adequate symptom relief. **Objective**: To assess the short-term (≥6 months) effectiveness of functional arthroplasty in reducing pain and improving QoL in patients with ADDwoR unresponsive to conservative therapies. **Methods**: A retrospective cohort study was conducted on 105 patients (median age 38 years, 80% female) treated at Santa Maria Hospital from January 2018 to December 2021. All patients had unilateral painful ADDwoR confirmed via MRI and underwent functional arthroplasty. Primary outcomes included pain reduction (VAS) and QoL improvements (QoL-TMJ questionnaire). Covariates such as age, gender, and baseline mood disturbances were analyzed for associations with surgical outcomes. Statistical analyses included the Wilcoxon rank test, Friedman’s ANOVA, and Spearman’s rank correlation. **Results**: Postoperative VAS scores significantly decreased (8.0 pre-op vs. 2.0 post-op, *p* < 0.001). QoL-TMJ scores improved significantly in pain (*p* < 0.001), activity (*p* < 0.05), mood (*p* < 0.001), and anxiety (*p* < 0.01), but no significant changes were observed in chewing and speaking functions. Improvements in QoL correlated strongly with pain reduction. Gender and age did not influence the outcomes, though females reported higher baseline mood disturbances. **Conclusions**: Functional arthroplasty effectively reduces pain and improves QoL in patients with ADDwoR, regardless of age or gender. However, limited improvement in chewing and speaking abilities highlights the need for targeted interventions. Future studies should assess the long-term outcomes to confirm the sustained benefits of this procedure.

## 1. Introduction

Temporomandibular joint disorders (TMDs) are one of the challenging pathologies to treat, with a higher prevalence in the female population, mainly in the fifth decade [1,2]. The most frequent symptoms of TMJ disorders are TMJ pain, headache, pain in the temporal and masseteric region, neck pain, tinnitus, vertigo, and limited jaw movements, particularly during chewing [3,4]. The etiology of TMDs includes trauma, parafunctional habits (such as bruxism and clenching), joint overloading, arthritides, psychological factors, and the ergonomic positioning of the head. The impact of psychological factors is difficult to calculate, but approximately 10% to 20% of patients with TMDs also manifest some form of anxiety (Axis II) [5]. Given the wide range of TMD symptoms and the difficulty in attributing a specific cause, TMDs are best understood as an interplay between joint pathology and psychological factors [6]. In the literature, different authors use different criteria to describe TMDs, so the reported prevalence of TMDs varies considerably. Among the adult population, the prevalence of signs and symptoms of TMDs is reported to be as high as 3% to 34% [7]. The main symptom of TMDs is TMJ pain, which could be associated with mechanical TMJ pathology, such as an inflammatory discal or retrodiscal status caused by disc displacement. Anterior displacement of the articular disc is the most common joint disorder [8]; the disc can be displaced even in other directions [9,10,11,12]. It can be reduced (ADDwR) or non-reduced (ADDwoR) with jaw opening. In the case of symptoms suggestive of ADDwoR, the most important radiological exam is MRI of the temporomandibular joint with a closed and open mouth [9,10,11,12]. Treatment modalities available for TMDs can be broadly classified into non-surgical, minimally invasive surgical, and open surgical procedures. The non-surgical treatment approaches include rest, counseling, and education of the patient [13], bite splints [14], physical therapy, and the regular use of non-steroidal anti-inflammatory medication (NSAIDs) [15] or injection of steroids [16] or botulinum toxin [17]. The minimally invasive surgical treatments include arthrocentesis and arthroscopy. Both these techniques involve the lavage of the superior compartment of the TMJ. Arthroscopy also involves the lysis of adhesions that may occur in the upper joint space and disc repositioning and fixation.

Patients who do not respond to the previously described techniques could be considered for surgical treatment. In the literature, different surgical techniques are described: discectomy [18,19], condylotomy [20], disc plication [21,22], disc repositioning [23], total joint prothesis [24], and functional arthroplasty [25]. The authors perform functional arthroplasty as a surgical technique for anterior disc displacement without reduction (ADDwoR) with persistent pain during jaw movement (jaw opening, chewing, lateral movements) and limited mouth opening (<40 mm) that has not responded to non-surgical and minimally invasive procedures. If left untreated, the articular disc may deform, degenerate, and progress to a more severe anterior displacement. Additionally, persistence of the internal derangement might produce condylar resorption with deformation and an overall decrease in condylar height [26]. The aim of this retrospective study is to evaluate quality of life and pain in patients with unilateral pain and ADDwoR unresponsive to non-surgical or minimally invasive techniques before and six months after functional arthroplasty using the VAS for pain assessment and a QoL-TMJ questionnaire. The QoL-TMJ questionnaire further investigates the patient’s pain, the main aspects of jaw functionality (such as chewing and speaking), the main aspects of daily living (such as general activities and recreational activities), and the main psychological elements that can be affected by TMJ disorders (such as mood and anxiety).

## 2. Materials and Methods

**Figure 1:**
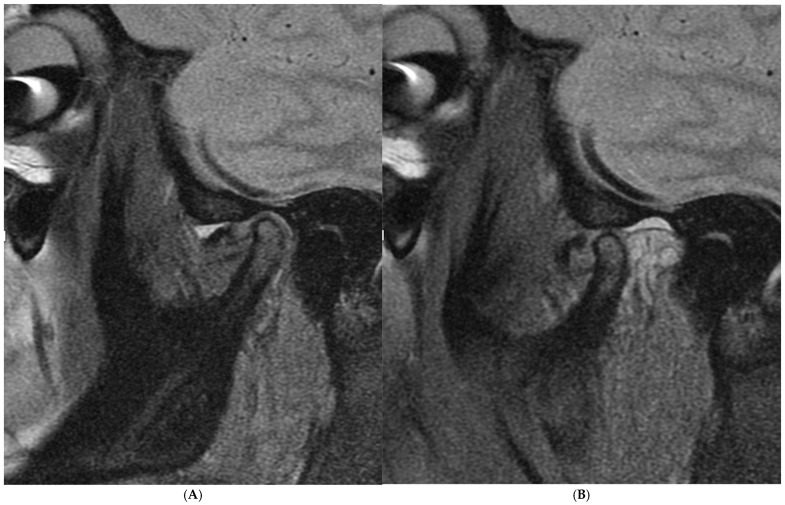
The picture shows an anterior disc dislocation with the mouth closed (**A**) and open (**B**) in MRI.

From January 2018 to December 2021, 203 patients with TMJ pain and limited jaw opening were admitted to Santa Maria Hospital in Terni with ADDwoR (anterior disc displacement without reduction). The diagnosis was confirmed by a 1.5 Tesla MRI, evaluating sagittal fat-suppressed T2W (FS-T2W) sequences with an open and closed mouth, in accordance with the diagnostic criteria (DC/TMD) [8,27] (Figure 1) and a clinical and radiological Wilkes classification [8,28].

The radiological diagnosis of DDWoR was confirmed not only by the radiologist but also by two surgeons (F.S. and V.V.), who analyzed the DICOMs using the Horos DICOM viewer v.4.0.0. Horos is a free and open-source code software (FOSS) program that is distributed free of charge under the LGPL license at Horosproject.org and sponsored by Nimble Co LLC d/b/a Purview in Annapolis, MD, USA.

All patients underwent functional arthroplasty surgery performed by the same surgical team.

The inclusion criteria were as follows:

- No previous diagnosis of other TMJ pathologies (trauma, rheumatologic and systemic diseases etc.);
- A diagnosis of unilateral ADDWoR, (according to the diagnostic criteria (DC/TMD) and Wilkes classification);
- A maximum assisted opening movement (MMO) < 40 mm;
- A record of the TMJ VAS score for pain assessment and the QOL TMJ questionnaire results before and at least six months after surgical treatment;
- Non-responsiveness to other non-invasive or minimally invasive techniques.

Out of the 203 patients admitted, 105 (51.7%) were included in the study, while 98 of them were not included (48.3%).

**Figure 2:**
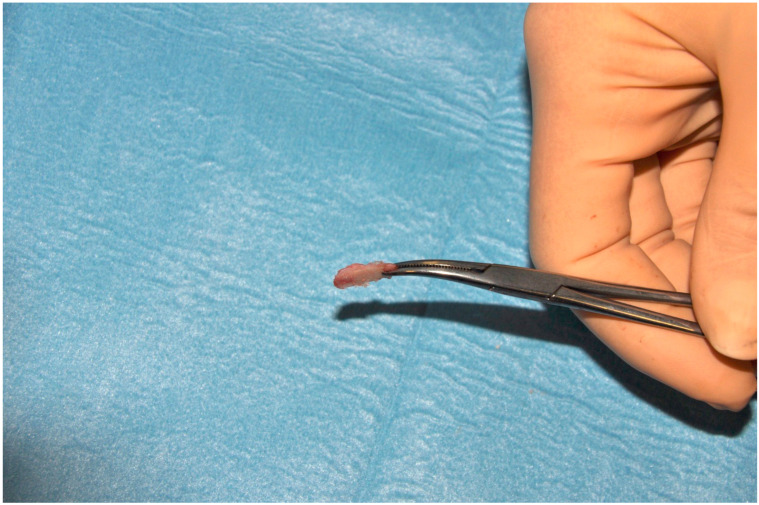
The picture shows the top of the condylar head, which has been removed for condylar remodeling. Condylar remodeling is performed using piezosurgery.

**Figure 3:**
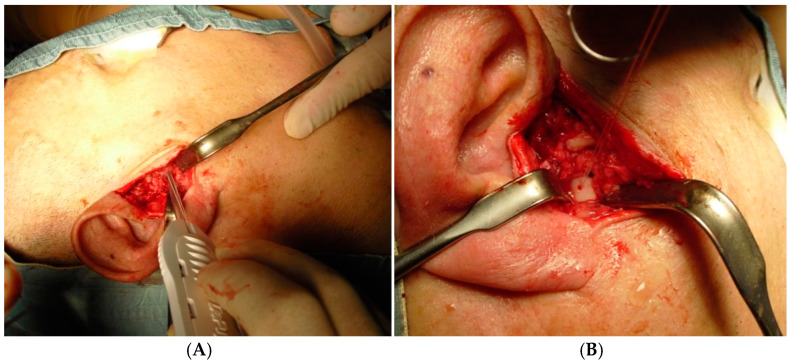
The pictures show the positioning of the Mitek Microfix QuickAnchor, which is sutured to the disc in order to restore its original position at the head of the condyle (**A**,**B**).

All patients underwent the same surgical procedure through an RHITNI (root of helix inter tragus notch incision), condylar head shaving (Figure 2), and disc repositioning with absorbable Mitek Microfix QuickAnchor Plus 1.3 (Figure 3), performed by the same surgeons (F.S and V.V.) [29,30,31,32].

The authors collected data from the hospital’s medical records. These data included the following: gender, age, nationality, medical comorbidities, symptoms, the visual analogic scale (VAS) score for TMJ pain assessment, the quality-of-life TMJ questionnaire (QoL-TMJ) before and six months after surgery, clinical signs, and radiological exams. The VAS assesses the patient’s TMJ pain on a scale from 0 to 10, where 0 indicates no pain and 10 represents the worst possible pain.

The TMJ-QoL, first proposed by Dimitroulis in 2010 [2], was based on the University of Washington QoL questionnaire for patients with head and neck oncology [33]. It was then adapted to patients with TMJ who were undergoing joint surgery and has previously been validated as an effective tool for evaluating the patient experience in relation to their TMJ-related quality of life post-surgery (Table 1) [2,34,35].

The first question (A) is about TMJ pain and the possible pain treatment with both NSAIDs and stronger analgesics like opioids. The following six questions (B to G) are about how the TMJ affects the patient’s daily activities, either involving the joint directly (chewing, talking) or indirectly (recreational and normal activities, and the feeling of bad mood and anxiety). The eighth question (H) investigates what part of the patient’s life was affected by the TMJ pain the month before surgery (the patient can circle up to three answers, which refer to the previous seven questions). The following three questions (I, J, K) refer to quality of life six months after surgery. These questions focus both on health-related well-being and psychological and social wellness. The last question (L) asks the patients whether they would recommend functional arthroplasty to friends and family members affected by the same TMJ pathology. The questions are answered on a 5-point scale (1 = excellent and 5 = poor) [24,36,37,38].

The work has been carried out in accordance with the Declaration of Helsinki. All procedures were performed within the relevant laws and institutional guidelines and have been approved by the Santa Maria Hospital Ethical Committee (TRAUMAX), 3993/19. All the patients who underwent functional arthroplasty surgery signed the informed consent form.

### Statistical Analysis

The sample size calculation was performed using a two-tailed paired t-test for the difference between two dependent means, with an effect size of 0.5, an alpha level of 0.05, and a power of 0.85 [38]. The calculation indicated that a minimum of 38 patients would be required to achieve adequate power.

Quantitative variables were analyzed for their distribution using the Kolmogorov–Smirnov test. Based on their asymmetrical distribution, they were represented by the median and interquartile range (IQR), both in the text and graphs. The comparison between two groups was performed using the Wilcoxon rank test, while the comparison among more than two groups was conducted using Friedman’s two-way ANOVA, followed by the Bonferroni post-hoc test. Correlation between scores was assessed using Spearman’s rank test.

Statistical analyses were performed using IBM SPSS Statistics version 29.0.1.0 (IBM, ARMONK, New York, NY, USA), and the graphs were created with GraphPad prism 10.0 (GraphPad software, Inc., La Jolla, CA, USA). The calculation for the sample size was performed using G*power 3.1.9.4., and *p* < 0.05 was considered statistically significant. Effect sizes were interpreted based on eta-squared (η^2^) thresholds: small (0.01), medium (0.06), and large (0.14).

## 3. Results

**Figure 4:**
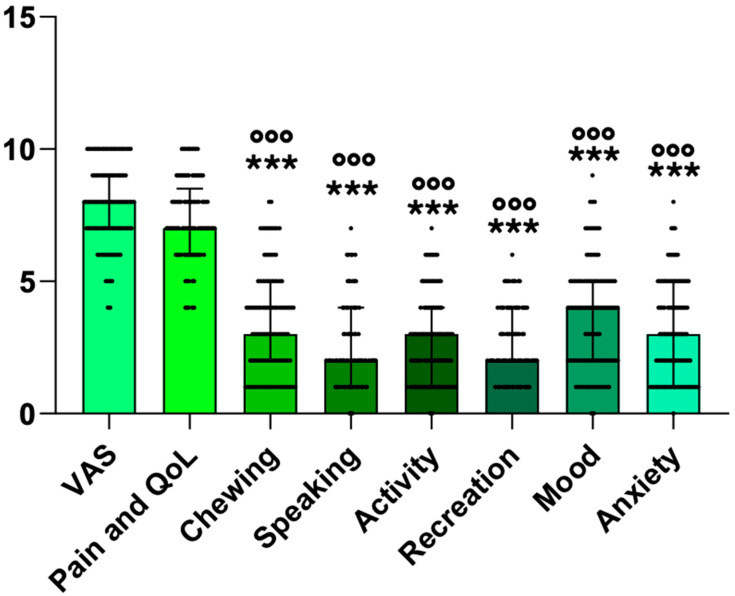
Scores of patients before surgery. The scatter plots with bars represent the median and interquartile range of scores before surgery. Statistical analysis was performed using Friedman’s two-way test with the Bonferroni post-hoc test. *** = *p* < 0.001 vs. VAS; °°° = *p* < 0.001 vs. pain and quality-of-life score.

A total of 105 patients with a median age of 38.0 (31.0–51.50) years were identified. A female prevalence (n = 84, 80%) with a male/female ratio of 1:4 was found. The analysis performed on scores before surgery showed that the VAS and pain and quality-of-life scores were significantly higher than all the other scores assessed (large effect size: η^2^ = 0.53) (Figure 4).

**Figure 5:**
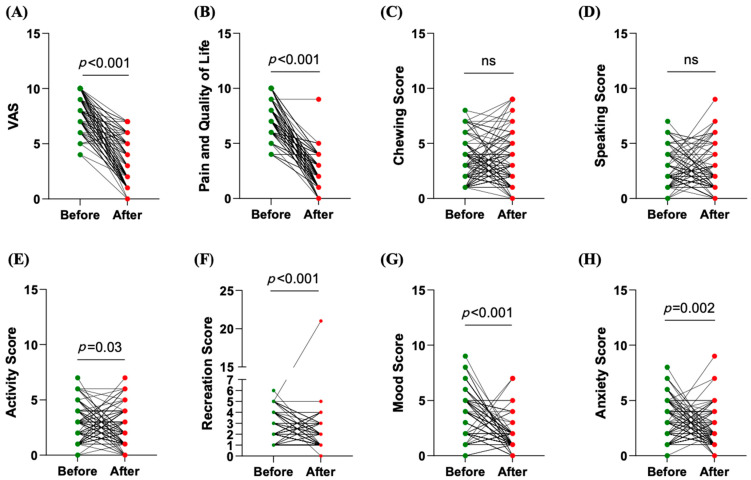
Comparison of scores before and after surgery. Before–after plots illustrate the distribution of pre- and post-surgical scores, highlighting a statistically significant difference in the VAS (**A**), pain and quality of life (**B**), chewing score(**C**), speaking score (**D**), activity score (**E**), recreation score (**F**), mood score (**G**), and anxiety score (**H**). The Wilcoxon test was applied.

Interestingly, comparing scores before and after surgery, the authors observed a reduction in pain assessed with the VAS (8.0 (7–9) vs. 2.0 (1.0–5.0), η^2^ = 0.73, *p* < 0.001) (Figure 5A), pain scores from the quality-of-life (QoL) questionnaire (7.0 (6.0–8.5) vs. 2.0 (1.0–3.0), η^2^ = 0.74, *p* < 0.001) (Figure 5B), activity scores (3.0 (1.0–4.0 vs. 2.0 (1.0–4.0), η^2^ = 0.04, *p* < 0.05) (Figure 5E), mood scores (4.0 (2.0–5.0) vs. 1.0 (1.0–2.0), η^2^ = 0.41, *p* < 0.001) (Figure 5G), and anxiety scores (3.0 (1.0–5.0) vs. 2.0 (1.0–3.0), η^2^ = 0.09, *p* < 0.01) (Figure 5H). It should be noted that a significant difference was also found for recreation scores (η^2^ = 0.18, *p* < 0.001), despite there being no change in the median value (Figure 5F). This suggests that, while the central tendency remained stable, individual responses varied significantly.

On the contrary, no differences were found for the chewing and speaking scores before and after surgery in our cohort (Figure 5C,D).

Spearman’s rank correlation analysis showed no correlation between scores before surgery. However, the same analysis performed only on the scores that were significantly reduced after surgery showed a slight but significant positive correlation between pain assessed with the VAS, pain scores from the QoL questionnaire, and mood scores (Table 2). In addition, a slight but significant negative correlation between the activity scores and both pain assessed with the VAS and pain scores from the QoL questionnaire as well as between the mood and recreation scores was detected (Table 2).

**Figure 6:**
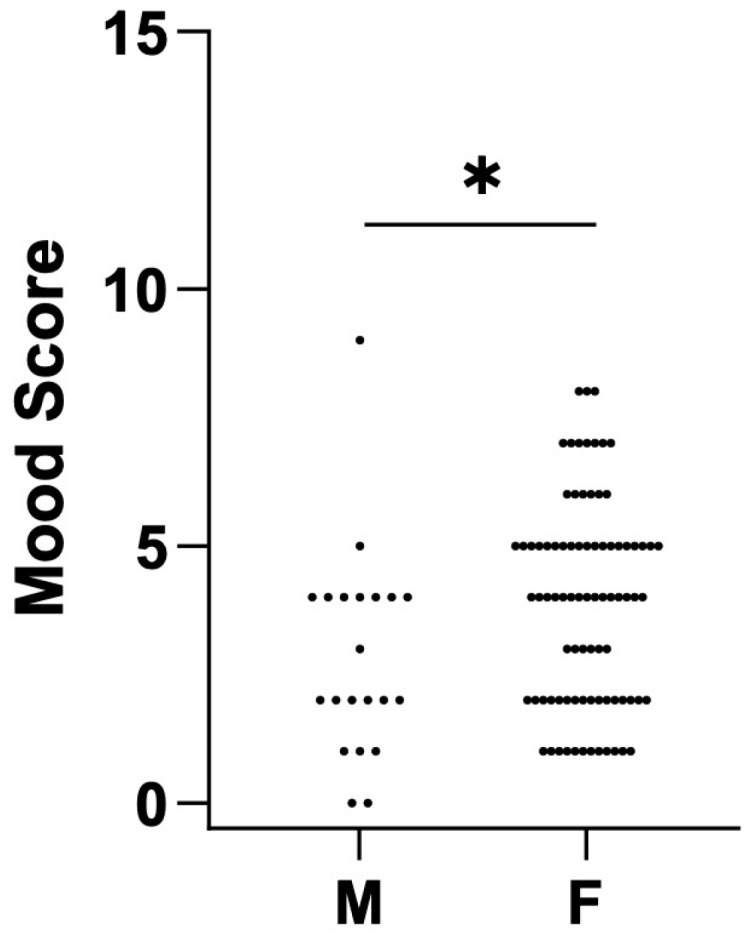
Worse mood scores for females before surgery. The scatter plot represents the distribution of mood scores for males and females before surgery. Statistical analysis was performed by the Wilcoxon test. * = *p* < 0.05.

No differences in scores were observed before and after surgery between patients aged ≤ 38 years and those aged > 38 years. Comparing females and males, we found a statistically significant difference at baseline in mood scores. Specifically, a higher score, corresponding to a worse mood, was seen in females compared to males (4 (2–5) vs. 2 (1.5–4), *p* < 0.05) (Figure 6). However, no differences were observed post-surgery.

## 4. Discussion

Anterior disc displacement without reduction (ADDwoR) of the temporomandibular joint (TMJ) often leads to persistent pain and reduced quality of life (QoL). Conservative treatments frequently fail to provide adequate symptom relief.

The authors aimed to assess the hypothesis that functional arthroplasty is a short-term (≥6 months) effective treatment for reducing pain and improving QoL in patients with ADDwoR unresponsive to conservative therapies.

The authors identified a total of 105 patients with a median age of 38.0 years. The cohort exhibited a significant female predominance (80%). This skewed gender distribution has already been confirmed in the literature [2,35,39].

The initial analysis focused on pre-surgery scores, revealing that pain assessed with the VAS and pain scores from the QoL questionnaire were significantly higher than all the other measured scores. This indicates that pain and its effect on quality of life were the most affected domains in these patients before undergoing surgery.

Upon comparing the scores before and after surgery, there was a marked reduction in several key areas. Substantial decreases were registered for pain assessed with the VAS and pain scores from the QoL questionnaire, highlighting the effectiveness of the surgical intervention in alleviating pain and improving the overall quality of life for these patients. Other scores also demonstrated significant improvements post-surgery, such as the activity, mood, and anxiety scores. In addition, the recreation score saw a significant but small reduction. This might be explained by the fact that a low score was already observed before surgery, underlining that these recreational activities are one of the areas less compromised in this type of patient. Surprisingly, the chewing and speaking scores did not show significant differences before and after surgery. It should be noted that these two scores, along with recreation scores, were among the lowest scores recorded before surgery. Nevertheless, this result suggests that surgical intervention did not impact these specific functions.

Spearman’s rank correlation analysis provided further insights into the relationships between different scores. Before surgery, no correlations were observed; however, after surgery, several slight but significant positive and negative correlations emerged. For instance, a positive correlation was found between pain assessed with the VAS and pain scores from the QoL questionnaire, indicating that patients who experienced a decrease in pain after surgery also reported an enhancement in their quality of life. Moreover, the mood score was found to be positively correlated with pain assessed with the VAS, demonstrating how the reduction in pain after surgery positively influenced mood. Interestingly, pain assessed with the VAS and pain scores from the QoL questionnaire negatively correlated with the activity score, highlighting how the reduction in pain and its effect on QoL also leads to an improvement in the ability to perform activities. These findings suggest interdependencies between different aspects of patient well-being, with improvements in one area being associated with improvements in another. However, a better mood was associated with a worse ability to perform daily activities.

The adopted surgical technique, named functional arthroplasty, is based on three principles: condylar shaving, disc repositioning, and fixation with the reconstruction of the lateral ligament. It is the authors’ opinion that condylar shaving produces the following results: (a) it eliminates the condylar irregularity observed in the case of long-lasting disc displacement; (b) it widens the articular space, which has been reduced by vertical height loss; (c) it creates scar adhesions between the disc and condyle, thus leading to greater stability post-surgery [29,30,31,32,39,40].

To the authors’ knowledge, this is the first study in the literature to assess improvements in quality of life after functional arthroplasty using the QoL-TMJ questionnaire.

Subjective methods to assess the patient’s perception of the clinical condition are fundamental to fully understanding the role of functional arthroplasty in improvements to the patient’s quality of life and general approach to daily living. Despite this, it is important to understand that pain perception and its impact on life differs among patients because of the pain threshold variability.

The main limitation of this study is the subjectivity of the answers, resulting from the fact that patients have different levels of adaptation, pain endurance, and resilience beyond the surgical technique used and the relatively small sample.

In conclusion, functional arthroplasty offers a robust solution for managing persistent TMJ pain and its associated impacts on daily life, paving the way for enhanced patient well-being. Future research should focus on long-term outcomes and further validate the sustained benefits of this surgical technique, as well as exploring strategies to address unchanged functional aspects like chewing and speaking.

## 5. Conclusions

This study demonstrates that functional arthroplasty is an effective surgical intervention for patients with anterior disc displacement without reduction (ADDwoR) who have not responded to non-surgical or minimally invasive treatments. The procedure significantly reduces pain, as evidenced by a marked decrease in pain (VAS score), and substantially improves overall quality of life, including key aspects such as mood, activity level, and anxiety.

Interestingly, despite the significant alleviation of pain and enhancement of life quality, the procedure did not impact chewing and speaking abilities, indicating these functions were less affected by the pathology or were possibly resistant to change post-surgery. The strong correlations observed between improvements in pain, mood, and quality of life underscore the interconnected nature of these domains, highlighting the comprehensive benefits of functional arthroplasty.

Notably, the intervention was equally effective across different genders and age groups, reinforcing its broad applicability. However, the study also revealed a higher baseline level of mood disturbance in females, a finding not widely discussed in the existing literature. This gender-specific difference emphasizes the need for tailored pre-surgical assessments and interventions.

## Figures and Tables

**Table 1:** TMJ qualify of life (TMJ-QoL) questionnaire.

Category	Rating	Description
A. PAIN	1	I have no pain.
	2	There is mild pain, but I do not need medication.
	3	I have moderate pain, which requires regular analgesics (e.g., paracetamol).
	4	I have severe pain controlled only by strong analgesics (e.g., Panadeine Forte).
	5	I have severe pain, which is not controlled by analgesics.
B. DIET AND CHEWING	1	I can chew and eat whatever I like.
	2	I can chew most things, except tough foods like steak and apples.
	3	I only stick to soft foods, such as pasta and soft bread.
	4	I need to cut all food into small pieces.
	5	I can only eat food that has been put through a blender.
C. SPEECH	1	My speech is normal.
	2	I have difficulty saying some words.
	3	I have difficulty in being understood over the telephone.
	4	Only my friends and family can understand me.
	5	I cannot be understood at all.
D. ACTIVITY	1	I am as active as I have ever been.
	2	There are times when I can’t keep up my old pace, but not often.
	3	I am often tired and have slowed down my activities, though I still get out.
	4	I don’t go out very often because I don’t have the strength.
	5	I am usually in bed or a chair and don’t leave home.
E. RECREATION	1	There are no limitations to recreation at home or away from home.
	2	There are a few things I can’t do, but I still get out and enjoy life.
	3	There are many times when I wish I could get out more, but I am not up to it.
	4	There are severe limitations to what I can do; mostly, I stay at home and watch TV.
	5	I can’t do anything enjoyable.
F. MOOD	1	My mood is excellent and unaffected by my TMJ disorder.
	2	My mood is generally good and only occasionally affected by my TMJ disorder.
	3	I am neither in a good mood nor depressed about my TMJ disorder.
	4	I am somewhat depressed about my TMJ disorder.
	5	I am extremely depressed about my TMJ disorder.
G. ANXIETY	1	I am not anxious about my TMJ disorder.
	2	I am a little anxious about my TMJ disorder, but I am coping.
	3	I am very anxious about my TMJ disorder and finding it difficult to cope.
	4	I am severely anxious about my TMJ disorder and not coping at all.
H. Issues Most on Your Mind (Circle up to 3 answers)	1	Nothing
	2	Pain
	3	Diet and chewing
	4	Speech
	5	Activity levels
	6	Recreation
	7	Mood
	8	Anxiety
I. Compared to the Month Before Surgery, How Would You Rate Your Overall Health-Related Quality of Life? (Post-Surgical Patients Only)	1	Much better
	2	Somewhat better
	3	About the same
	4	Somewhat worse
	5	Much worse
J. How Would You Rate Your Health-Related Quality of Life Over the Past Month?	1	Excellent
	2	Very good
	3	Good
	4	Fair
	5	Poor
K. How Would You Rate Your Overall Quality of Life Over the Past Month (Considering Factors Such as Family, Friends, Spirituality, and Leisure Activities)?	1	Excellent
	2	Very good
	3	Good
	4	Fair
	5	Poor
L. If a Relative or Friend Had TMJ Problems Similar to Yours, Would You	1	Recommend TMJ surgery as the primary treatment
	2	Recommend TMJ surgery only if other measures (e.g., physiotherapy, splint therapy, medications) fail
	3	Recommend TMJ surgery only as a very last resort
	4	Not recommend TMJ surgery at all

**Table 2:** Results of Spearman’s rank correlation analysis of the relationship between scores after surgery.

Spearman’s Rho Correlation	VAS	Pain and Quality-of-Life Score	Activity Score	Recreation Score	Mood Score
Pain and Quality of Life score	*ρ* = 0.19 *η*^2^ = 0.04 *p* = 0.046	/	/	/	
Activity score	*ρ* = − 0.21 *η*^2^ = 0.04 *p* = 0.028	*ρ* = −0.23 *η*^2^ = 0.05 *p* = 0.018	/	/	
Recreation score	*ρ* = −0.004 *p* = 0.965	*ρ* = −0.09 *p* = 0.342	*ρ* = −0.05 *p* = 0.581	/	
Mood score	*ρ* = 0.36 *η*^2^ = 0.13 *p* < 0.001	*ρ* = −0.16 *p* = 0.090	*ρ* = −0.002 *p* = 0.984	*ρ* = −0.22 *η*^2^ = 0.05 *p* = 0.024	
Anxiety score	*ρ* = −0.1 *p* = 0.324	*ρ* = −0.20 *p* = 0.053	*ρ* = 0.09 *p* = 0.376	ρ = 0.05 *p* = 0.628	*ρ *= −0.12 *p* = 0.205

*ρ* = correlation coefficient; *η*^2^ = Eta-squared.

## Data Availability

Data is unavailable due to privacy.

## Abbreviations

The following abbreviations are used in this manuscript:

ADDwoR	anterior disc displacement without reduction
IQR	interquartile range
MMO	maximum assisted opening movement
QoL	quality of life
